# Corrigendum: Chemotaxis towards autoinducer 2 mediates autoaggregation in *Escherichia coli*

**DOI:** 10.1038/ncomms13979

**Published:** 2016-12-15

**Authors:** Leanid Laganenka, Remy Colin, Victor Sourjik

Nature Communications 7: Article number: 12984; DOI: 10.1038/ncomms12984 (2016); Published: 09 30 2016; Updated: 12 15 2016

Two previous studies describing the effects of autoinducer 2 (AI-2) on biofilm formation and chemotaxis in *Escherichia coli* were inadvertently omitted from the reference list of this Article. A reference to these studies along with ref. 27 should have been provided in the Introduction, as follows: ‘It has been previously reported that, in *E. coli*, AI-2 can modulate biofilm formation (González-Barrios *et al.* 2006) and virulence-related phenotypes such as chemotaxis, swimming motility and attachment to host cells *in vitro* (Bansal *et al.* 2008, ref. 27).' These findings should have also been mentioned in the Abstract, where the physiological role of AI-2 in *E. coli* and other bacteria is referred to, and in the sections discussing motility, chemotaxis and biofilm formation in *E. coli*.

González Barrios, A. F. *et al.* Autoinducer 2 controls biofilm formation in *Escherichia coli* through a novel motility quorum-sensing regulator (MqsR, B3022). *J. Bacteriol.* **188,** 305-316 (2006).

Bansal, T., Jesudhasan, P., Pillai, S., Wood, T. K. & Jayaraman, A. Temporal regulation of enterohemorrhagic *Escherichia coli* virulence mediated by autoinducer-2. *Appl. Microbiol. Biotechnol.* **78,** 811-819 (2008).

