# Supplementary material for: Chemotaxis towards autoinducer 2 mediates autoaggregation in Escherichia coli
Source: Nat Commun. 2016 Sep 30;7:12984. doi: 10.1038/ncomms12984 (PMC5056481; doi:10.1038/ncomms12984)
Supplement: Supplementary Information — Supplementary Figures 1-12, Supplementary Table 1 and Supplementary References. [file ncomms12984-s1.pdf]

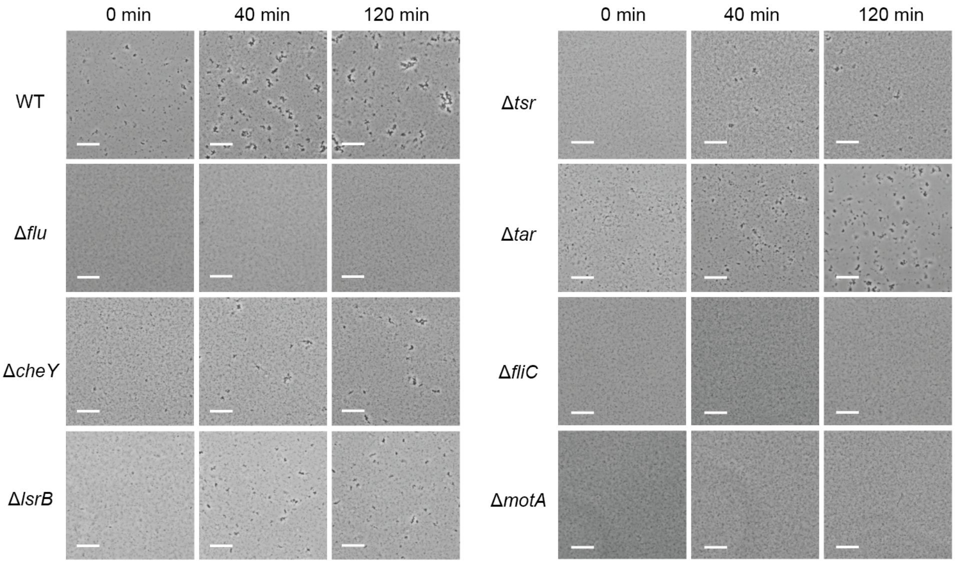

Supplementary Figure 1 | Autoaggregation of *E. coli* W3110 in presence of native Ag43 phase variation depends on Ag43, motility, chemotaxis and AI-2 sensing. Cells were grown to OD_{600} of 0.6 at 37°C and aggregation was assayed at room temperature and OD_{600} of 2.0 using microscopy as described in Methods. Scale bars, 20 μm.

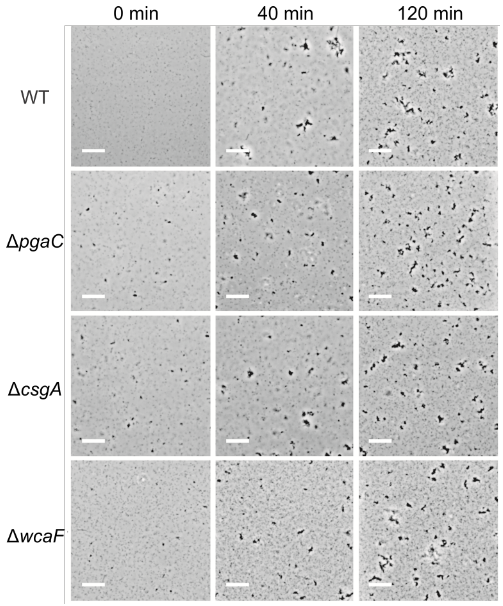

Supplementary Figure 2 | Autoaggregation of *E. coli* W3110 in presence of native Ag43 phase variation does not depend on major biofilm matrix components. Aggregation of strains deficient in production of poly-β-1,6-N-acetyl-D-glucosamine (*pgaC*), curli (*csgA*) or colanic acid (*wcaF*) production was analysed as in Supplementary Figure 1. Scale bars, 20 μm.

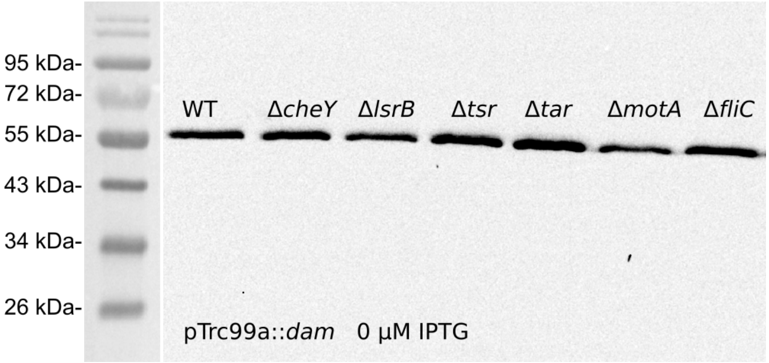

Supplementary Figure 3 | Immunoblot analysis of Ag43 expression by the strains used in this study. All strains expressed Dam methyltransferase at basal level from *trc* promoter without IPTG induction. Immunoblotting was performed as described in Methods and Ag43 staining with specific antibody was detected using chemiluminescence. Molecular weight marker (left) on the same membrane was imaged using reflected light.

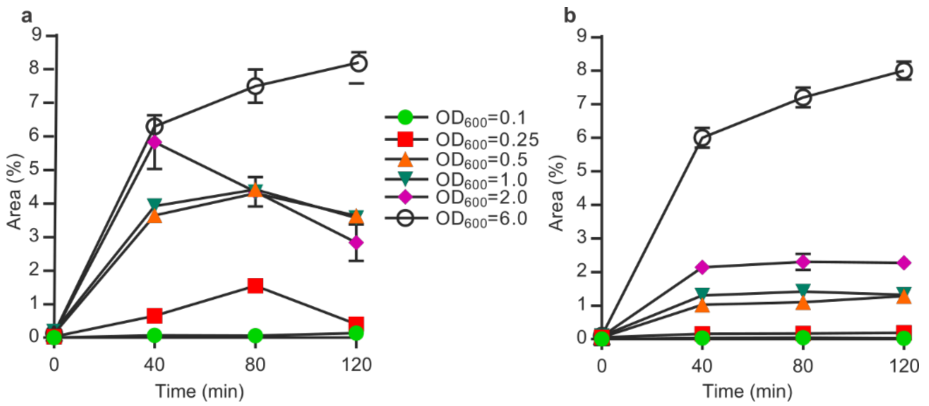

Supplementary Figure 4 | Time course of aggregation in dependence of optical density of the cell culture. Cultures grown as in Figure 1 were resuspended in fresh TB medium to indicated optical densities. **a,** Wild type. **b,** Δ*cheY*. Error bars indicate standard deviation of three independent replicates.

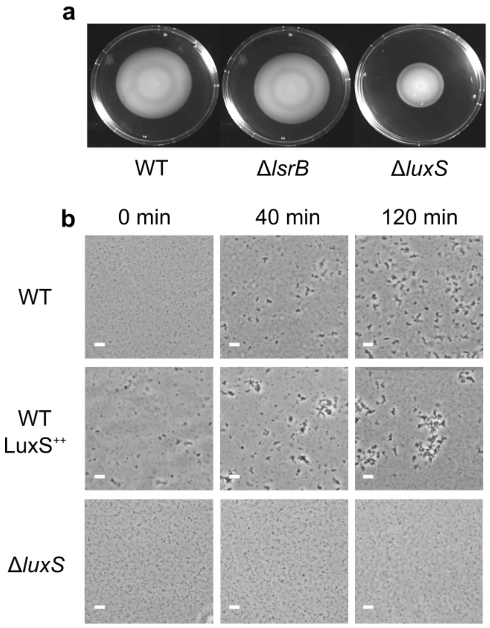

Supplementary Figure 5 | AI-2 production affects motility and aggregation. a, Motility of Δ *lsrB* and Δ *luxS* mutants compared to the wild-type, tested on 0.3% soft agar TB plates where spreading of *E. coli* requires motility and chemotaxis. **b,** Effects of *luxS* overexpression and deletion on aggregation. Scale bars, 20 µm.

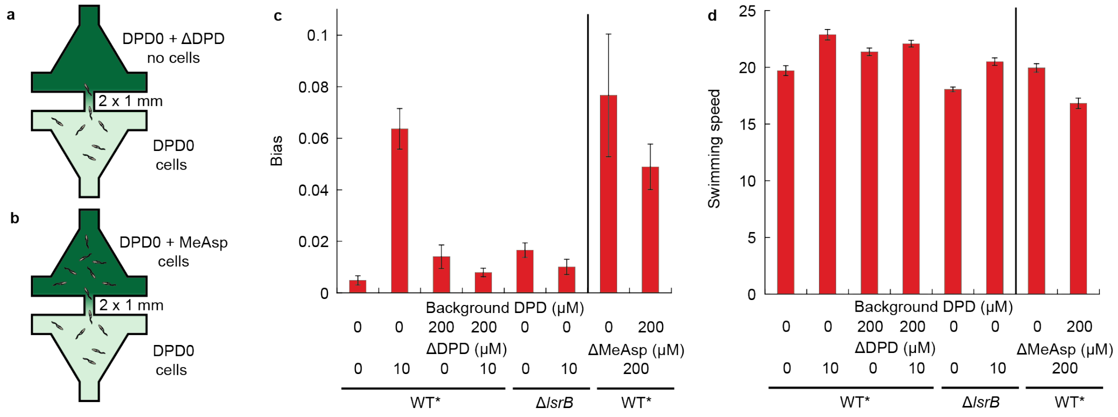

Supplementary Figure 6 | Chemotaxis towards and in presence of AI-2. a, Schematic representation of the top view of the microfluidic device used to measure chemotaxis towards metabolized synthetic DPD/AI-2. Cells are added only in the lower concentration reservoir, and measurements are performed in the middle of the channel linking the two reservoirs. **b,** Same as in (a) but for device used to measure chemotaxis towards non-metabolized attractant MeAsp, where cells are added on both sides. **c,** Chemotactic bias of Δ*flu* (wild-type for chemotaxis, marked as WT*) or Δ *lsrB* towards DPD/AI-2 (left) or MeAsp (right) in presence of either no or 200 µM background concentration of DPD/AI-2. Δ*flu* was used to avoid complications with precise quantification of chemotaxis in presence of aggregate formation. Measurement was done using DDM (see Methods) one hour after loading the device. **d,** Average swimming speed of the cells, measured by DDM, for the same experiments as (c). In (c) and (d), the error bars represent measurement errors.

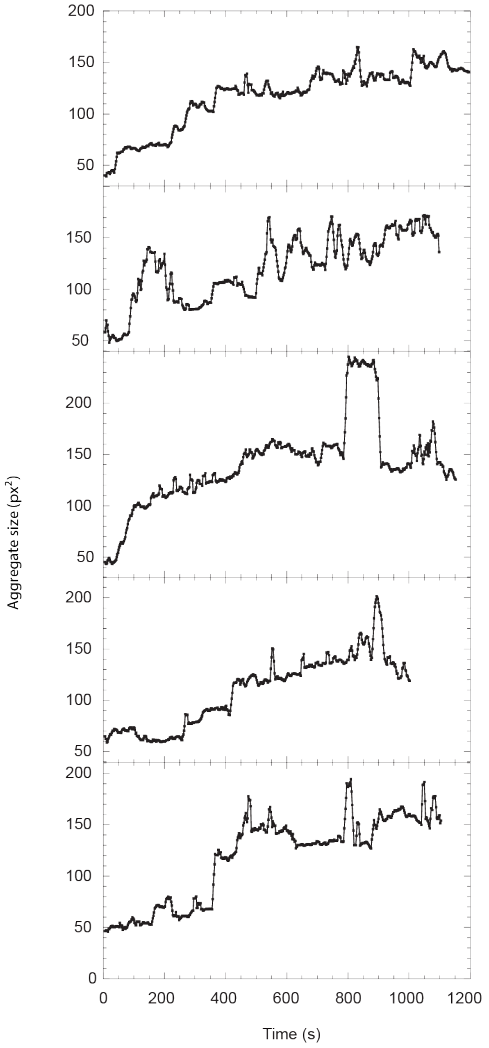

Supplementary Figure 7 | Size of individual wild-type aggregates as a function of time. Aggregate sizes were quantified in time-lapse movies, acquired as in Figure 3a and shown in Supplementary Movie 1.

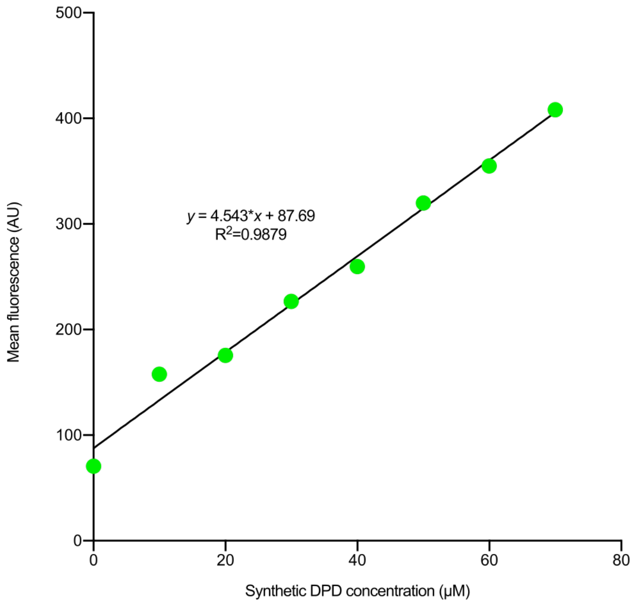

Supplementary Figure 8 | Calibration of *lsr* reporter using synthetic DPD/AI-2. Calibration was performed as described in Methods. Data are means of three experiments; error bars, indicating standard deviation of three independent replicates, are smaller than the symbol size. Note that this calibration might underestimate reporter response to AI-2 in case when only a fraction of DPD spontaneously converted to AI-2.

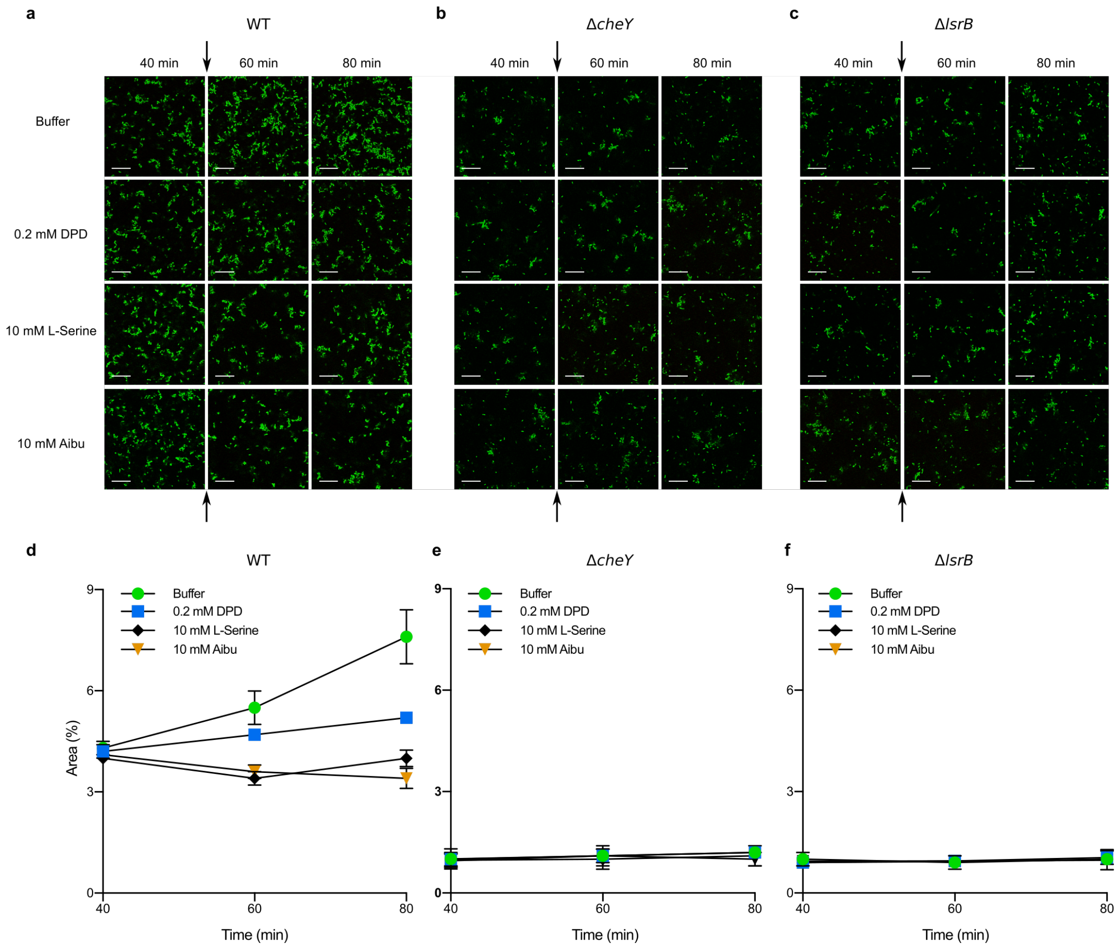

Supplementary Figure 9 | Effects of added DPD/AI-2, L-serine or 2-aminoisobutyric acid on pre-formed aggregates. a-c, Autoaggregation of the wild type, Δ *cheY* and Δ *lsrB* cells, measured in 8-well glass bottom plates. After 40 min of autoaggregation, 40 µl of the tethering buffer (control), DPD/AI-2, L-serine or 2-aminoisobutyric acid (AIbu, nonmetabolizable analogue of L-serine) were added as indicated. Scale bars, 20 µm. **d-f,** Quantification of cell aggregation in the microscopic images. Means of three independent replicates are shown; error bars indicate standard deviation.

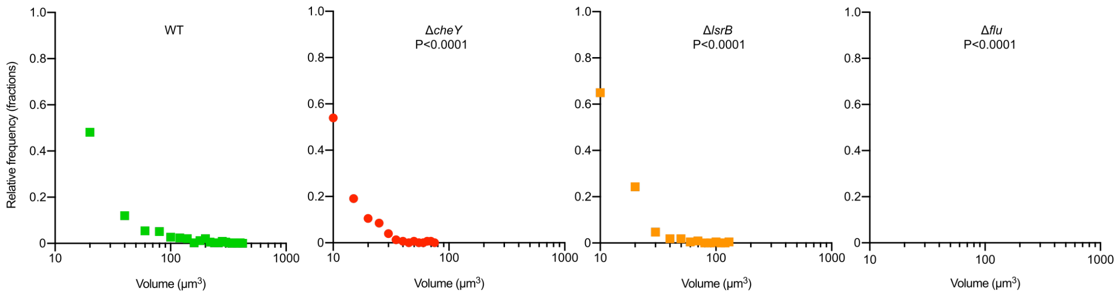

Supplementary Figure 10 | Distribution of the microcolony volumes within static biofilms shown in Figure 5c. Quantification of the microcolony sizes was done as described in Methods. No identifiable microcolonies were formed in Δ*flu* strain. *P* values were calculated using unpaired *t*-test (data distribution was confirmed to be normal). The difference between size distribution of Δ *cheY* and Δ *lsrB* microcolonies was not significant.

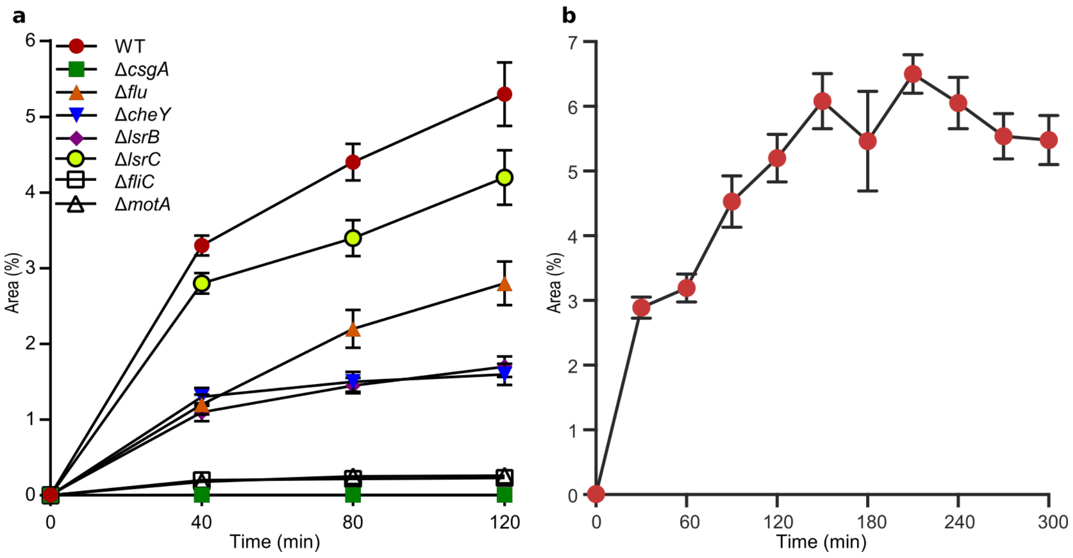

Supplementary Figure 11 | Quantification of curli-mediated aggregation. a, Quantification of experiments shown in Figure 6a, for cells grown at 30°C to OD_{600} of 1.0. **b,** Quantification of aggregation on longer time scale (compare to Figure 3d for cells grown at 37°C). Error bars indicate standard deviation of three independent replicates.

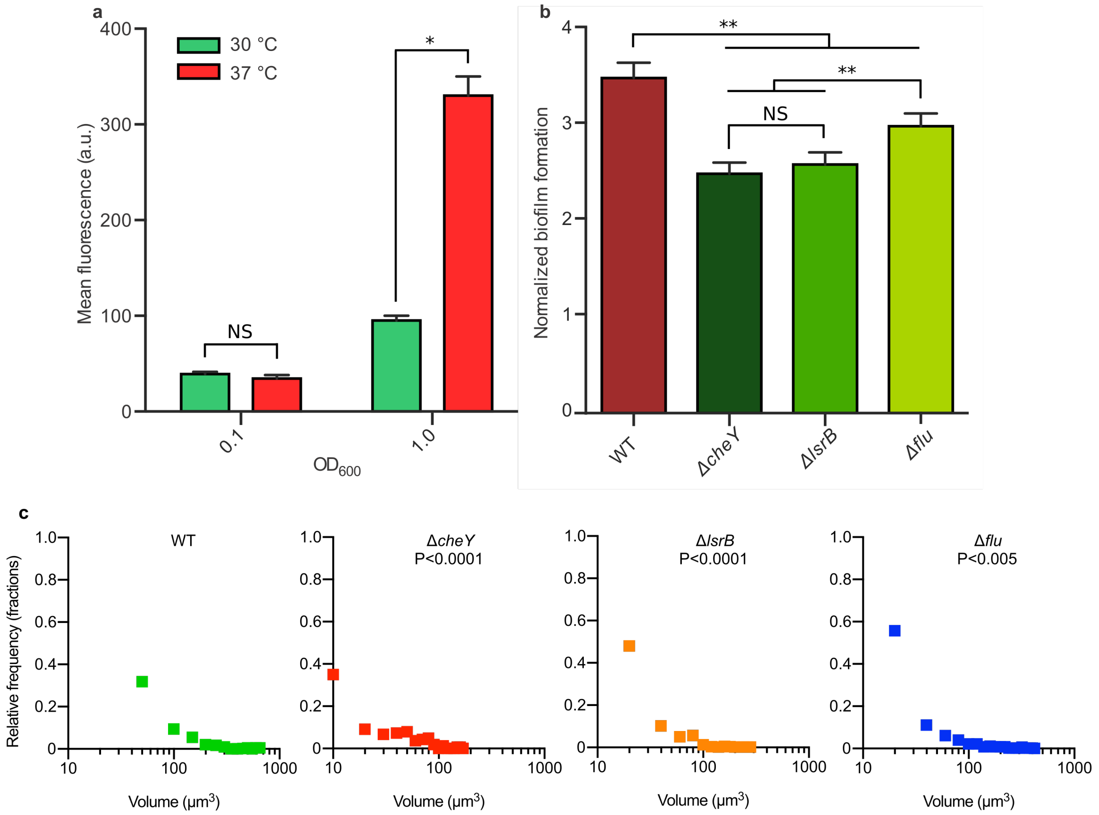

Supplementary Figure 12 | Production of AI-2 and effects of AI-2 chemotaxis and Ag43 on biofilm formation at 30°C. a, Activity of the *lsr* operon at 30°C and 37°C and at indicated optical densities, measured using flow cytometry and expressed in arbitrary units (AU) of fluorescence. *P* values were calculated using Mann-Whitney test (*P=0.05, NS-not significant, P>0.05). **b,** Biofilm formation for 30°C static culture at 24 h, quantified by CV staining. Shown in arbitrary units (AU) are CV values normalized by the optical density. *P* values were calculated using Mann-Whitney test (** P<0.05, NS – not significant, P>0.05). Error bars indicate standard deviation of three (a) and five (b) independent replicates. **c,** Distribution of the microcolonies' volumes within static biofilms shown in Figure 6b. Quantification and analysis was done as in Supplementary Figure 10. The difference between size distribution of Δ *cheY* and Δ *lsrB* microcolonies was not significant.

Supplementary Table 1 | *E. coli* strains and plasmids used in this study.

Strains	Relevant genotype or phenotype[a]	Reference
W3110 (RpoS$^+$)	W3110 derivative with functional RpoS (*rpoS396(Am)*)	1
VS670	W3110 Δ*motA*::Km^r	This work
VS683	W3110 Δ*fliC*::Km^r	This work
VS695	W3110 Δ*cheY* Km^s	This work
VS703	W3110 Δ*pgaC* Km^s	This work
VS704	W3110 Δ*csgA*::Km^r	This work
VS705	W3110 Δ*wcaF*::Km^r	This work
VS823	W3110 Δ*luxS* Km^s	This work
VS824	W3110 Δ*flu* Km^s	This work
VS825	W3110 Δ*lsrB* Km^s	This work
VS826	W3110 Δ*tsr* Km^s	This work
VS827	W3110 Δ*tar* Km^s	This work
VS828	W3110 Δ*lsrC* Km^s	This work
**Plasmids**		
pTrc99a	Amp^r; Expression vector; pBR ori; pTrc promoter, IPTG-inducible	2
pUA66	Km^r; Expression vector; SC101 ori; GFPmut2 under control of promoter of interest	3
pVS1515	Amp^r; *egfp* in pTrc99A, IPTG-inducible	This work
pVS1721	Amp^r; *luxS* in pQE30, IPTG-	4

	inducible	
pVS1722	Amp^r; *dam* in pTrc99A, IPTG-inducible	This work
pVS1723	Km^r; P_{lsr}-*egfp* in pUA66	This work
pCP20	Amp^r; *flp*	[5]

[a]Amp^r, Km^r indicate ampicillin and kanamycin resistance, respectively.

Supplementary References

1. Serra, D. O., Richter, A. M., Klauck, G., Mika, F. & Hengge, R. Microanatomy at cellular resolution and spatial order of physiological differentiation in a bacterial biofilm. *MBio* **4,** e00103–13 (2013).

2. Amann, E., Ochs, B. & Abel, K.J. Tightly regulated tac promoter vectors useful for the expression of unfused and fused proteins in *Escherichia coli*. *Gene* **69**, 301–315 (1988).

3. Zaslaver, A. *et al.* A comprehensive library of fluorescent transcriptional reporters for *Escherichia coli*. *Nat. Methods* **3,** 623–8 (2006).

4. Schauder, S., Shokat, K., Surette, M.G. & Bassler, B.L. The LuxS family of bacterial autoinducers: biosynthesis of a novel quorum-sensing signal molecule. *Mol. Microbiol*. **41**, 463–276 (2001).

5. Cherepanov, P. P. & Wackernagel, W. Gene disruption in *Escherichia coli*: TcR and KmR cassettes with the option of Flp-catalyzed excision of the antibiotic-resistance determinant. *Gene* **158,** 9–14 (1995).
